# Awareness Development and Usage of Mobile Health Technology Among Individuals With Hypertension in a Rural Community of Bangladesh: Randomized Controlled Trial

**DOI:** 10.2196/19137

**Published:** 2020-12-07

**Authors:** Yasmin Jahan, Md Moshiur Rahman, Abu S G Faruque, Mohammod Jobayer Chisti, Kana Kazawa, Ryota Matsuyama, Michiko Moriyama

**Affiliations:** 1 Graduate School of Biomedical and Health Sciences Hiroshima University Hiroshima Japan; 2 International Centre for Diarrhoeal Disease Research, Bangladesh Dhaka Bangladesh

**Keywords:** mobile health, hypertension, behavior changes, awareness development, lifestyle, Bangladesh

## Abstract

**Background:**

Hypertension (HTN) is a major modifiable risk factor and the leading cause of premature deaths globally. The lack of awareness and knowledge have been identified as risk factors in low- and middle-income countries including Bangladesh. Recently, the use of mobile phone SMS text messaging is found to have an important positive impact on HTN management.

**Objective:**

The study aimed to develop awareness and knowledge in order to enhance lifestyle behavior changes among individuals with HTN in a rural community of Bangladesh by using health education and mobile health (mHealth) technology (SMS text messaging).

**Methods:**

A prospective randomized 5-month intervention, open-label (1:1), parallel-group trial was implemented among the individuals with HTN aged 35 years or older. Both men and women were included. Between August 2018 and July 2019, we enrolled 420 participants, selected from a tertiary level health facility and through door-to-door visits by community health workers. After block randomization, they were assigned to either the intervention group (received SMS text messaging and health education; n=209) or the control group (received only health education; n=211). The primary outcome was the evaluation of self-reported behavior changes (salt intake, fruits and vegetables intake, physical activity, and blood pressure [BP], and body weight monitoring behaviors). The secondary outcomes were measurements of actual salt intake and dietary salt excretion, blood glucose level, BP values, and quality of life (QOL).

**Results:**

During the study period, a total of 8 participants were dropped, and the completion rate was 98.0% (412/420). The adherence rates were significantly higher (9%) among the control group regarding salt intake (*P*=.04) and physical activity behaviors (*P*<.03), and little differences were observed in other behaviors. In primary outcome, the focused behavior, salt intake less than 6 g/day, showed significant chronological improvement in both groups (*P*<.001). The fruits intake behavior steadily improved in both groups (*P*<.001). Participants in both groups had a custom of vegetables intake everyday/week. Physical activity suddenly increased and continued until the study end (*P*<.001 in both groups). Both BP and body weight monitoring status increased from baseline to 1 month but decreased afterward (*P*<.001). In case of secondary outcomes, significant chronological changes were observed in food salt concentration and urinary salinity between the groups (*P*=.01). The mean systolic BP and diastolic BP significantly chronologically decreased in both groups (systolic BP, *P*=.04; diastolic BP, *P*=.02.*P*<.05). All of these supported self-reported behavior changes. For the QOL, both groups showed significant improvement over the study periods (*P*<.001).

**Conclusions:**

Based on these results, we suggest that face-to-face health education requires integration of home health care provision and more relevant and timely interactive SMS text messages to increase the effectiveness of the intervention. Besides, community awareness can be created to encourage “low-salt culture” and educate family members.

**Trial Registration:**

Bangladesh Medical Research Council (BMRC) 06025072017; ClinicalTrials.gov NCT03614104; https://clinicaltrials.gov/ct2/show/NCT03614104 and UMIN-CTR R000033736; https://tinyurl.com/y48yfcoo

**International Registered Report Identifier (IRRID):**

RR2-10.2196/15523

## Introduction

Noncommunicable diseases (NCDs) are the leading cause of death and disability globally. Each year an estimated 41 million people die from NCDs, accounting for about 70% of total mortality worldwide [1]. Among the NCDs, hypertension (HTN) is a major modifiable risk factor and the leading cause of premature death [2]. The global prevalence of HTN is projected to increase up to 29.2% by 2025 (or >1.5 billion people) [3-5]. Currently, the prevalence of HTN [3] among adults is higher in low- and middle-income countries (31.5% or 1.04 billion people) than in high-income countries (28.5% or 349 million people) [6]. A recent report suggested that 18% or 12 million adults aged 25 years or older suffer from HTN in Bangladesh [7].

The risk factors for developing HTN are more salt intake, sedentary lifestyle, excess weight, unhealthy diet, tobacco consumption, and chronic stress [8-10]. The World Health Organization recommends salt intake of 5 g/day for adults in order to reduce blood pressure (BP) and risk of cardiovascular diseases [11], whereas Bangladeshi people consume more than 17 g salt/day [12].

According to the NCD risk factors survey, the prevalence of self-reported HTN is 12.5% (men 10.9% and women 13.9%) and one-third of the Bangladeshi population never measured their BP due to lack of opportunity and accessibility to the health care system [13]. Despite the increasing prevalence of HTN, a substantial proportion of the population remains undiagnosed due to lack of awareness to seek medical care and high treatment costs [7,11,14]. Controlling BP at a population level may be associated with a decrease in cardiovascular diseases regardless of the increasing burden of chronic diseases such as kidney disease and diabetes [15]. The comorbid conditions related to HTN may cause the greatest morbidity burden if left untreated, especially in low-resource settings [3,4]. Therefore, continuous and long-term BP monitoring including medication adherence is needed [16,17]. So far, different types of nonpharmacological interventions have been developed and tested with aims to improve BP control [18].

In this regard, the 2-way SMS text messaging communication is a commonly used approach that can help develop awareness and improve an individual’s self-management about HTN [19]. Nowadays, the mobile phone has become the favored device to communicate and gather information together worldwide [19]. Mobile phone–based interventions could address individual-level factors in health by facilitating timely patient access to relevant health information and support, making patient–provider communication easier, and providing context-specific support that translates into action [19]. Moreover, SMS text messaging is available on all mobile phone service providers that support 1- or 2-way communication [20]. As an example, some trials suggested that SMS text messaging reminders may improve behavior, clinic attendance, attention in care, and self-reported medication adherence [21]. Studies have explored the efficacy of SMS-based interventions for people with HTN to improve treatment compliance and BP control [22,23]. Although the results of trials suggested the potential for small to moderate effect sizes in improving compliance and BP control, the effects were not statistically significant [20]. Furthermore, systematic reviews and meta-analysis on SMS text messaging–based interventions provide researchers useful insight into the most promising applications of SMS text messaging in supporting health care, disease prevention, and case management. Despite the general interest in mobile phone–based interventions related to disease management and behavior change, the allied evidence remains limited [20]. However, while a significant number of studies focused on chronic disease prevention, particularly cardiovascular disease prevention by utilization of SMS text messaging, or on diabetes management [19], there are limited studies on BP outcomes [24].

To fill the gap, in this study, we provided periodic reminders via SMS text messaging along with in-person health education aimed at increasing awareness about BP level and behavior changes to positively influence patients’ perceptions of HTN and their adherence to treatment remedies [25]. Participants from the rural community were selected for the study based on 3 criteria: (1) have lack of awareness (understanding) about HTN due to low health literacy, (2) do not have sufficient health infrastructure, and (3) lower adherence rate compared to urban community people [7,26]. Hence, to expand awareness of the target population, 3 innovative techniques (Portable Health Clinic, food and urine salinometer, and SMS text messaging) were introduced in this study [27].

The purpose of the study was to develop awareness and knowledge in order to enhance lifestyle behavior changes among individuals with HTN in a rural community of Bangladesh by using mobile health (mHealth) technology (SMS text messaging) and in-person health education. Thus, this approach could minimize the hypertensive status of the participants.

## Methods

### Study Design

This was a single-center, prospective randomized (1:1), open-label, parallel-group study conducted in a rural community of Bangladesh involving people with HTN. The intervention period was 5 months, and the total study duration was 12 months. The detailed study design has been previously reported [27]. The study is reported according to the Consolidated Standards of Reporting Trials of Electronic and Mobile HEalth Applications and onLine TeleHealth V1.6.1 (Multimedia Appendix 1) [28].

### Study Population and Sampling

This study was conducted between August 2018 and July 2019, and we enrolled a total of 420 participants aged between 35 and 71 years. Individuals with HTN were identified from a tertiary level health facility by reviewing the registered clinician’s prescriptions and from the hospital logbook. Further, community health workers (CHWs) who received information from the hospital did a door-to-door visit to select participants. The female CHWs were selected from the study community and trained for data collection, including providing in-person health education and SMS text messaging. A purposive sampling method was used for enrolling participants who met the eligibility criteria and were approached to participate voluntarily in the study. Participants (either sex) enrolled in this study who were diagnosed with HTN were aged 35 years or older, had 1-5 years of schooling, resided within a radius of 3 miles from the tertiary hospital, decided to stay in the community for at least five months, had a personal cell phone or access to a shared phone, could exchange views freely, and were willing to participate in the study. Individuals with mental illnesses or serious comorbidities such as malignancy or tuberculosis were excluded from this study.

### Sample Size

The sample size calculation was based on behavior changes of the study participants and has been described previously [27]. The sample size was calculated with a 2-tailed 5% significance level, 80% power with a CI of 95% (1–α), and to detect varying differences in the effectiveness of the 2 intervention groups. Adherence rates were assumed to be differing between 10% and 12% with 90% in the study group with a presumption that 6% of the participants would be lost during follow-up. Thus, considering the largest size of the calculated sample sizes, the study finally had a sample size of 210 in each group.

### Randomization

A randomization schedule was prepared following a permuted block randomization technique using a block size of 4 based on a computer-generated series of numbers. The serially numbered sealed envelopes were used to allocate the participants to either the intervention (n=209) or the control (n=211) group after written informed consent was obtained.

### Study Procedure

The study procedure is described in the following sections and was also described in detail previously [27] (also see Multimedia Appendix 2).

### Intervention Group

The intervention group received 5 months’ in-person health education along with a health education booklet and SMS text messaging to develop awareness and knowledge, and motivate them for behavior changes, with the content of both educational materials and SMS text messaging being the same.

We developed health education materials, SMS text messaging, and questionnaire according to the Dietary Approaches to Stop Hypertensive (DASH) diet and the National Institutes of Health– and the World Health Organization–recommended guidelines [29]. All materials were translated to Bangla for the participants. The SMS text messaging served as a reminder for behavior changes based on the DASH diet. The contents of the SMS text messages included physical activity, medication adherence, less salt intake (ie, salt intake <6 g daily), eat more fruits and vegetables and less beef and mutton, avoid junk foods, do physical activity for 30 minutes at least five days a week, and take antihypertensive medication regularly [27]. The contents of SMS text messages are shown in Multimedia Appendix 3.

At the time of enrollment, CHWs visited the participant’s household in the morning. The CHWs conducted interview and performed physical examination (height, weight, and BP) and blood test (random blood sugar [RBS]) of the study participants at the same time. Furthermore, the CHWs checked food salinity by measuring salt in liquid foods and urinary salinity (spot test [only at baseline] and first-morning urine [overnight urine]). They also checked the urinary protein levels. Afterward, the CHWs provided in-person health education to change participant’s behaviors. Participants of the intervention group were followed up every month up to 5 months (two times in the first month and one time for the rest). The SMS text messages were sent 5 times for the first month and one time a week for the remaining 4 months (a total of 21 SMS text messages).

### Control Group

The control group received the same health education booklet as the intervention group at the time of enrollment. They were followed up similarly every consecutive month up to 5 months (two times in the first month and one time for the rest). However, they received only in-person health education identical to that of intervention group and not the SMS text messages.

### Outcome Measures

The primary outcome was the evaluation of self-reported lifestyle behavior changes (salt intake, fruits intake, vegetables intake, physical activity, BP monitoring, and body weight monitoring). The measurement tool was a Likert-type response scale and evaluated lifestyle behavior changes every month on a scale from 1 to 5, where 1 demonstrates “0 days/week (approximately 13-14 g daily salt intake)” in case of salt intake, or “0 days/week” in case of fruits intake, vegetables intake, physical activity, or “Never/month” (BP and body weight monitoring)” and 5 implies the “Everyday/week (<6 g daily salt intake),” “Everyday/week” (fruits intake, vegetables intake, physical activity), or “8 times/month” (BP and body weight monitoring).

Regarding salt intake, CHWs asked participants to fill their spoons with the salt they usually used and measured approximately how many grams they were taking per day. In case of fruits and vegetables intake, consuming a single piece of fruit and at least one meal with vegetables everyday/week corresponds to the Likert-type response scale score of 5. However, the quantities of fruits and vegetables intake were not measured. With regard to physical activity, participants performing at least 30 minutes of physical activity everyday/week were given a score of 5 on the Likert-type response scale. The detailed content of the behavior change questionnaire is presented in Multimedia Appendix 4. The behavior change was measured by adherence rates of each behavior which was operationally defined as the improvement of behavior (ie, any shift to the higher score on the Likert-type response scale in case of each behavior) at 5 months compared with that at the baseline.

The secondary outcomes were (1) actual salt intake measured by food salinity checker (TANITA electron salinometer SO-313) and dietary salt excretion measured by urine (KME-03, KOUNO ME Institute) [30]. We measured the salt concentration of food using 3 different categories (yellow indicated moderate [0.4%-0.7%], green indicated thick [0.8%-1.1%], and red indicated very thick [1.2%–1.4%] considering 100 mL of food); BP values; RBS level; urinary protein excretion measured by urinalysis strip test for checking comorbidities (ie, kidney diseases and diabetes). The strip analysis usually detects protein concentration in urine. The results were graded as negative, trace (10-20 mg/dL), 1+ (30 mg/dL), 2+ (100 mg/dL), 3+ (300 mg/dL), or 4+ (1000 mg/dL) [29]. The quality of life (QOL) was measured using the standardized EuroQol-5 dimensions-5-level (EQ-5D-5L) questionnaire, which was developed for measuring 5 dimensions (mobility, self-care, usual activities, pain/discomfort, and anxiety/depression) [31,32] and each dimension has 5 levels: no problems, slight problems, moderate problems, severe problems, and unable to/extreme problems. A higher score indicated better QOL [33]. In this study, we set the standard value (tariff) for EQ-5D-5L to estimate the impact of health care interventions on QOL (used Japanese region’s tariff) [34].

### Ethical Consideration

This study was approved by the Bangladesh Medical Research Council (BMRC; Approval No. 06025072017) and registered with the Clinical Trial Registry (NCT03614104). The UMIN Registration Number is R000033736. This study was conducted in accordance with the Declaration of Helsinki and the Ethical Guidelines on Clinical Studies of the Ministry of Health, Labor and Welfare of Japan.

All participants had been explicitly explained about the objectives, importance, risks, and benefits of the research before recruitment. Participation was completely voluntary and written informed consent was obtained from all participants.

### Quality Control

The data quality control has been explained in detail in our previous work [27].

### Statistical Analysis

In order to examine the effectiveness of the intervention, baseline and end-line surveys were conducted in both study groups. The intention-to-treat analysis was conducted to compare the outcomes of the intervention and control groups. To ensure the comparability of the randomized samples, all baseline indicators at the time of registration were analyzed for both groups. The data were expressed as the mean (SD) or median (range) and cross-tabulation for the categorical variables and as frequencies and percentages for the discrete variables. The baseline differences in categorical variables between the groups were examined using the chi-square (χ^2^) test and for continuous variables using the *t* test or Mann–Whitney *U* test, as appropriate.

For the primary outcome, we have tested the difference between adherence rates of the intervention and control groups for each behavior. The adherence rate (proportion) was calculated as follows: dividing the number of individuals who adhered to the intervention (or control) group by the number of participants in the intervention (or control) group in the 5 months (ie, participants who dropped out from the program were not included in the denominator). Statistical differences were tested by the χ^2^ test.

In addition, to evaluate the effect of SMS text messaging plus in-person health education compared to only in-person health education on the participant’s behavior, we employed 2 types of statistical model: one assumed that there was no effect of the intervention on the time-series change of the proportion of each Likert-type response scale score, and the other assumed that the intervention affected the time-series change of the proportion of each Likert-type response scale score. In other words, we fit a multinomial logit model to observe the proportional change of each Likert-type response scale score (1) with only the time (baseline, 0.5 months, 1 month, 2 months, 3 months, 4 months, and 5 months) as an explanatory variable (time-only model, which means the chronological effect of intervention did not differ from that of control), and (2) with the time and the type of group (intervention/control) as explanatory variables (time–group model, which means the chronological effect of the intervention was different from that of control). The detailed information about the model structure is described in Multimedia Appendix 5. In the time–group model, the control group was used as the reference and the proportional effects of the intervention were estimated. Except for vegetables intake behavior, we fit the models for segmented times designed for each behavior because the changes in each behavior were not constant (Figure S2 and Tables S1 and S2 in Multimedia Appendix 5). The segmentation was implemented by assuming the constant trends (1) from baseline to 0.5 months, and 0.5 months to 5 months in salt intake and physical activity, and (2) from baseline to 0.5 months, 0.5 month to 1 month, 1 month to 2 months, and 2 months to 5 months in BP and body weight monitoring. We evaluated the goodness of fit for both models by the likelihood ratio test with a significance level (α=.05). In that case, the result of the likelihood ratio test was not significant when we selected the time-only model. In case the time–group model was selected as the better fit model, the relative effects of an intervention on the control group were calculated from the estimated coefficients with 95% confidence intervals (CIs). To obtain maximum likelihood estimates of parameters, we used the statistical software R (R Foundation) [35] and ‘multinom’ function implemented in the package ‘nnet’ [36]. For data manipulation and graphical presentation, we used the packages ‘dplyr’ [37] and ‘ggplot2’ [38]. The strength of association was measured by calculating the risk ratios and their 95% CIs. The Friedman test was performed for the chronological comparison of each behavior.

For secondary outcomes, to compare numeric outcome variables between the 2 groups, 2-way repeated-measures ANOVA was performed after verification of data normality. Moreover, the number of participants for each salt concentration measurement was counted at every month, and the Friedman test was performed.

The wealth index was constructed using principal component analysis of the household assets. The households were classified into socioeconomic status quintiles based on the wealth index: quintile (poor, lower middle, middle, upper middle, and rich).

Data were analyzed using the statistical software packages SPSS for Windows version 25.0 (IBM) and Epi Info version 7.0 (CDC). Two-tailed *P*-values were used, and the level of significance of association was considered at <.05.

### Data Availability

The data supporting the findings of this study are available from the corresponding author upon reasonable request.

## Results

### Study Population

**Figure 1:**
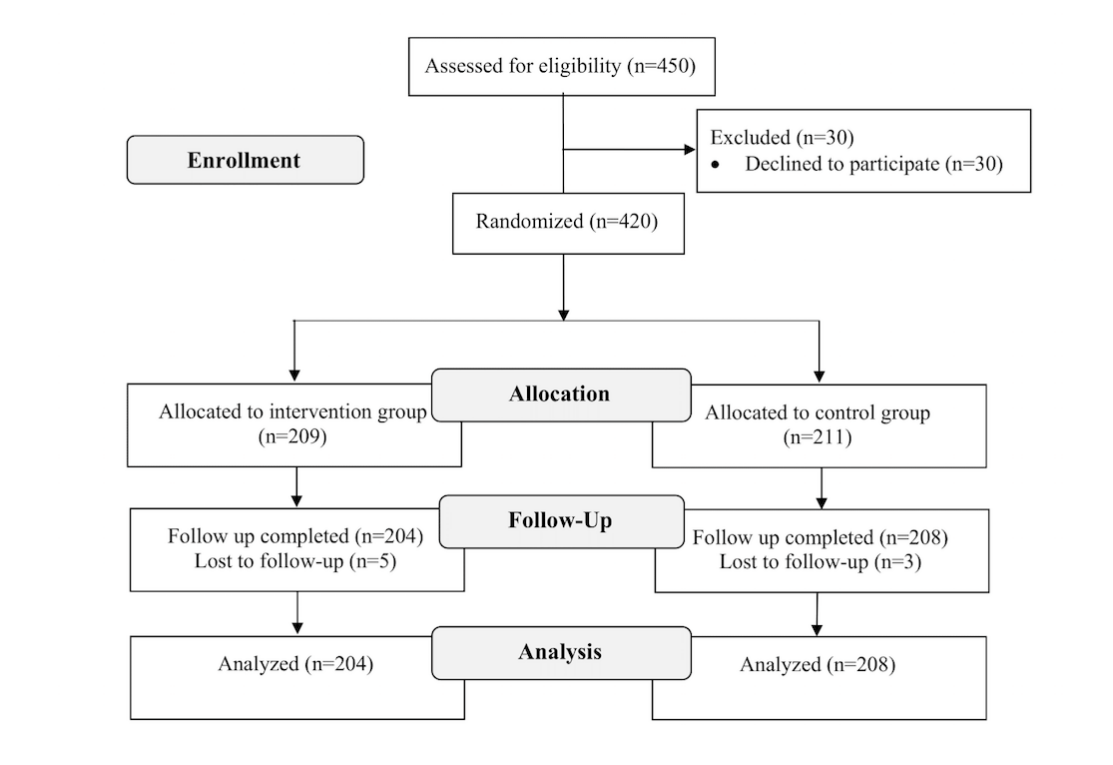
Flow chart of the study participants.

Of the 450 individuals who met the eligible criteria, 420 consented to participate in the study (consent rate, 93.3%). During the intervention period, 5 from the intervention group and 3 from the control group dropped out due to the refusal to continue. Therefore, 204/209 (97.6%) and 208/211 (98.6%) completed the program in the intervention and control groups, respectively, and their data were analyzed (Figure 1).

### Demographic Characteristics

The demographic data and participant profiles of each group are shown in Table 1. In total, the mean (SD) age was 47.1 (8.4) years. As much as 361/420 (86.0%) of the participants were female, and 346/361 (95.8%) were housewives. Most participants had their spouses (378/420 [90.0%]), and 80/420 (19.0%) had an educational background of secondary schooling completed or higher. Moreover, 287/420 (68.3%) of participants were taking their antihypertensive medicine regularly. Regarding tobacco use, 110/420 (26.2%) of the total study participants smoked either smokeless tobacco or cigarettes or both, whereas only 17/420 (4.0%) smoked cigarettes every day. With regard to RBS levels, the average (SD) value was calculated and compared to the intervention group (9.3 [SD 5.5]) and the control group (8.9 [SD 4.8]). As much as 113/204 (55.4%) of participants from the intervention group and 117/208 (56.3%) from the control group had normal or lesser values than those recommend for RBS. Only 4 participants had 1+ (30 mg/dL) and 2 participants had 2+ (100 mg/dL) urinary protein excretion among the total study participants. No statistically significant differences were observed between the 2 groups (Table 1).

**Table 1:** Demographic characteristics, behavior, and comorbidities of the study participants (N=420).

Variables name		Intervention group (N=209)	Control group (N=211)	*P* value
**Age (in years)**				.10^a^
	Mean (SD)	46.4 (8.3)	47.8 (8.6)	
	Range	35-70	35-71	
**Gender, n (%)**				.26^b^
	Female	184 (88.0)	177 (83.9)	
**Educational status, n (%)**				
	Primary complete	97 (46.4)	104 (49.3)	.33^b^
	Secondary incomplete	68 (32.5)	71 (33.6)	
	Secondary complete or higher	44 (21.1)	36 (17.1)	
**Marital status, n (%)**				
	Married	190 (90.9)	188 (89.1)	.09^b^
**Occupation, n (%)**				
	Housewife	177 (84.7)	169 (80.1)	.46^b^
**Wealth index, n (%)**				
	Poor	46 (22.0)	59 (28.0)	.56^b^
	Lower middle	53 (25.4)	53 (25.1)	
	Middle	58 (27.8)	46 (21.8)	
	Upper middle	44 (21.1)	45 (21.3)	
	Rich	8 (3.8)	8 (3.8)	
**Antihypertensive drug, n (%)**				
	Take regularly	151 (72.2)	136 (64.5)	.09^b^
**Tobacco use^d^ (Everyday), n (%)**				
	Smoke cigarette	5 (2.4)	12 (5.7)	.08^b^
	Smokeless tobacco intake	53 (25.4)	57 (27.0)	.23^b^
	Comorbidities			
**Random blood sugar (mmol/L)**				
	Mean (SD)	9.3 (5.5)	8.9 (4.8)	.80^c^
	Normal range (4.4-7.8) or below	113 (26.9)	117 (27.9)	.77^b^
**Urinary protein (n)**				
	Trace amount	1	0	.56^b^
	1+	3	1	
	2+	1	1	

^a^*t* test.

^b^Chi-square test.

^c^Mann–Whitney *U* test.

^d^Duplicate answer.

### Primary Outcome (Behavior Change)

#### Adherence Rate

The adherence rates for the intervention and control groups are presented in Table 2. The adherence rates were significantly higher among the control group participants regarding salt intake behavior (the difference was 9% between the 2 groups; *P*=.04) and physical activity behavior (also 9%; *P*=.03); however, very little differences were observed in other behaviors.

**Table 2:** Adherence rates in the intervention and the control groups.

Behavior	Adherence rate^a^		Differences	χ^2^ test
	Intervention group, %	Control group, %	Intervention – Control	*P* value
Salt intake	66.5	75.8	–9.3	.04
Fruits intake	64.6	66.8	–2.2	.68
Vegetables intake	1.0	0.0	1.0	.25
Physical activity	72.7	82.0	–9.3	.03^b^
Blood pressure monitoring	12.4	13.3	–0.8	.88
Body weight monitoring	1.4	1.4	0.0	>.99

^a^Proportion of behavior improved (number of participants who improved their behavior from baseline to 5 months).

^b^*P* value <.05.

#### Intervention Effects on Behavior Changes

**Figure 2:**
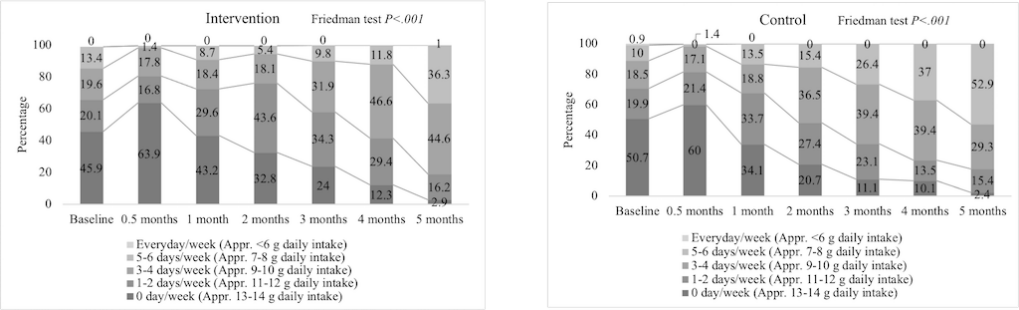
Chronological salt intake behavior changes between the intervention and control group.

Regarding behavior changes, the salt intake behavior which we recommended (ie, <6 g daily intake) showed significant improvement in both the intervention and control groups (*P*<.001). The intervention group improved from “0 days/week” (coded as 1) to “5-6 days/week” (coded as 4) from 96/209 (45.9%) at baseline to 6/209 (2.9%) at 5 months and 28/204 (13.4%) at baseline to 74/204 (36.3%) at 5 months, respectively, over the study period. Besides, the control group extensively improved from “0 days/week” to “5-6 days/week” from 107/211 (50.7%) at baseline to 5/208 (2.4%) at 5 months and 21/211 (10.0%) at baseline to 110/208 (52.9%) at 5 months, respectively (Figure 2). The chronological changes in other lifestyle behaviors (fruits intake, vegetables intake, physical activity, BP monitoring, and body weight monitoring) are described in Multimedia Appendix 5 and the respective figures are shown in Multimedia Appendix 6 (Figures S3-S7).

The fruits intake steadily improved in both groups, which was statistically significant (*P*<.001 in each group). In both groups, the percentages were increased by around 20% among those participants who ate fruits everyday/week from baseline to the end of the study. Participants in both groups had a custom of vegetable intake everyday/week. There were no changes between the 2 groups, and hence the differences were not significant.

The majority of the participants (140/209 [67.0%] in the intervention and 163/211 [77.3%] in the control group) did not indulge in physical activity at baseline. However, after the intervention, most of them (195/204 [95.6%] in the intervention and 197/208 [94.7%] in the control group) reached the scale “Everyday/week” and continued to take part in some kind of physical activity on a daily basis until the study end (*P*<.001, both groups).

BP and body weight monitoring status per month were increased from baseline to 1 month but decreased afterward. The difference was statistically significant in both groups (*P*<.001). Up to 1 month, CHWs measured BP and body weight two times per month, but afterward one time a month for remaining 4 months (Multimedia Appendix 6).

In accordance with the results of likelihood ratio tests, we selected the time–group model for estimating salt intake (*P*<.001) and fruits intake behavior (*P*<.001; Table 3). Besides, the time–group model was not selected for physical activity, BP, and body weight monitoring as the changes in these behaviors were inconsistent. These results showed that the difference between the effect of intervention on the 2 groups was significant in salt intake and fruits intake than other behaviors.

The intervention consistently contributed to the increase of the Likert-type response scale scores 2 (1-2 days/week) and 5 (Everyday/week) in fruits intake. The relative effect of the intervention ranged from 1.28 (95% CI 1.00-1.61) times increase in score 2 (1-2 days/week) to 2.24 (95% CI 1.68-2.97) times increase in score 5 (Everyday/week). By contrast, in case of salt intake, the intervention resulted in a decrease of the Likert-type response scale scores 3 (approximately 9-10 g daily intake) and 4 (approximately 7-8 g daily intake) in term 2. The relative effects were estimated as 0.63 (95% CI 0.50-0.79) times decrease in score 3 (approximately 9-10 g daily intake) and 0.29 (95% CI 0.22-0.39) times decrease in score 4 (approximately 7-8 g daily intake; Multimedia Appendix 5).

**Table 3:** Statistical significance in the likelihood ratio test and the selected model for each behavior among the study groups.

Behavior	*P* value	Selected model
Salt intake	<.001	Time–group
Fruits intake	<.001	Time–group
Vegetables intake	—	—
Physical activity	.36	Time only
Blood pressure monitoring	.41	Time only
Body weight monitoring	.32	Time only

### Secondary Outcomes

**Figure 3:**
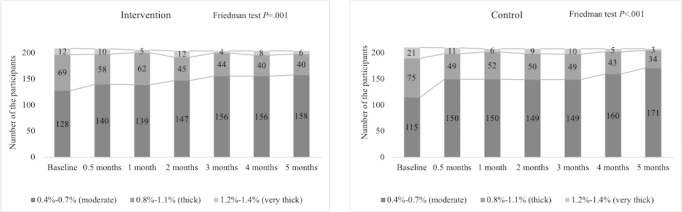
Food salt concentration changes among the intervention and control groups.

Our study findings showed significant chronological changes in urinary salt concentration between the groups (*P=*.01; Table 4). In the case of food salinity, the salt concentration appeared as thick (0.8%-1.1%) and very thick (1.2%-1.4%), and gradually decreased from the baseline to the study end, with statistically significant changes observed between the 2 groups. Especially, the control group showed an ample drop (*P*<.001), compared to the intervention group (*P*=.001; Figure 3). Conversely, the evaluation of urinary salt excretion from the previous day’s salt intake showed inconsistent changes which decreased at the study end. However, mean value of urinary salt excretion did not decrease from 10 g/day at any point.

Systolic BP (SBP) and diastolic BP (DBP) were significantly chronologically decreased in both groups (*P*<.001) over the study period. The mean SBP and DBP dropped between the groups over the study period, more in the intervention group, and the changes were statistically significant (*P*=.04 and *P*=.02, respectively).

In the case of QOL, both groups showed significant improvement at the end of the study (*P*<.001). The intervention group improved more compared to the control group, and a significant difference was observed (*P*<.001) between the groups. However, we did not find any significant interaction between the 2 groups (Table 4).

**Table 4:** Chronological changes in the secondary outcomes between the 2 groups.

Measurement and group																		
		Values							Two-way repeated measures ANOVA									
		Baseline^a^	0.5 months	1 month	2 months	3 months	4 months	5 months	Within groups				Between groups		Interaction			
									*F* value			*P* value	*F* value	*P* value	*F* value		*P* value	
**Urine salinity (g)^b^, mean (SD)**																		
	Intervention group (N=204)	8.04 (2.71)	10.36 (3.11)	10.71 (2.69)	10.85 (2.74)	10.75 (2.80)	10.59 (2.82)	10.18 (2.84)	1.91		.09		2.97	.01	1.62	.15		
	Control group	8.23 (3.05)	10.93 (2.88)	10.58 (2.94)	10.78 (3.20)	10.75 (3.05)	10.58 (3.20)	10.30 (3.21)										
**Systolic blood pressure (mmHg), mean (SD)**																		
	Intervention group (N=204)	136.9 (19.2)	130.0 (16.7)	128.4 (17.1)	127.6 (15.4)	127.9 (17.5)	127.2 (15.7)	125.7 (16.1)	9.95		<.001		4.42	.04	1.7	.12		
	Control group (N=208)	136.9 (19.3)	132.4 (16.3)	132.3 (16.2)	131.7 (16.1)	132.1 (17.2)	131.6 (16.7)	128.3 (15.4)										
**Diastolic blood pressure (mmHg), mean (SD)**																		
	Intervention group (N=204)	89.4 (9.9)	86.3 (9.3)	84.6 (9.0)	84.9 (8.3)	85.3 (9.3)	85.1 (8.4)	84.4 (8.0)	7.71		<.001		5.82	.02	0.38	.89		
	Control group (N=208)	90.5 (10.4)	87.9 (9.6)	87.0 (9.7)	86.5 (9.5)	87.5 (10.3)	87.4 (10.0)	86.1 (9.3)										
**Quality of life (EQ-5D-5L score), mean (SD)**																		
	Intervention group (N=204)	0.70 (0.10)			0.76 (0.11)			0.75 (0.09)	21.58		<.001		17.83	<.001	0.13	.88		
	Control group (N=208)	0.71 (0.10)			0.76 (0.11)			0.75 (0.09)										

^a^Baseline comparison was tested in each item and no significant differences were observed.

^b^Baseline data of urinary salt excretion was excluded from analysis because it was collected from spot urine, whereas we have collected the overnight urine from the rest of the months.

## Discussion

### Principal Findings

Among the study population, no significant differences were found in baseline demographic characteristics, comorbidities, and medication use as well as behavior as a primary endpoint and BP values, salinity, and QOL as secondary endpoints. Therefore, block randomization was successful. Completion rates of both groups were high and the drop-out rate was only 2%, whereas in previous lifestyle behavior studies the drop-out was 10%-20% [39,40].

In order to make this study more comprehensive, at baseline, we incorporated the RBS and urinary protein investigations as HTN’s comorbidities in the analysis. The baseline data estimated the high prevalence of diabetes. Research revealed that more than 80% of comorbidities are associated with HTN where controlling BP is difficult [41]. Therefore, not only behavior change education, but also comprehensive disease management strategies such as dietary change, medication adherence may be required to control HTN. We thus suggest future research to take these issues into consideration.

We expected that SMS text messaging could enhance the effects of behavior changes more than in-person health education alone (10%-12% difference). We thought periodic, weekly SMS text messaging would act as a reminder to change, which can improve self-reported behavior. Nevertheless, the results of this study did not support the expected assumption. The reason for this may perhaps be that relevant, timely, and convenient SMS text messaging can be more effective [42]. As an example, a timely, interactive telephonic behavior change intervention was found to be significantly effective for heart failure patients to prevent re-admission [23].

We supposed that SMS text messaging could be a useful tool for inducing behavior changes in Bangladesh as well as in other low- and middle-income countries due to their fragile health infrastructure [43]. However, we were not able to show the additional effect on in-person health education in the intervention group. In fact, a better improvement was found in the control group compared to the intervention group. The possible reasons may be the following: (1) mHealth technology needs prompt feedback and continued monitoring [44], but in this study we only sent weekly SMS text messages; (2) participants may not have had time to compose the response or may not have had credit on their phones (SMS text messaging needs a small amount of money [credit] to send messages) [33]. Some participants may have reacted as intended but thought that it was not necessary to respond; (3) the conciseness of our SMS text messages may have been difficult to understand (ie, reduce salt intake to 1 teaspoon size); and (4) our SMS text messages were generalized, and more tailored SMS text messages for individuals may have improved the effectiveness of the intervention.

Rather, in-person health education without SMS text messaging was more effective. Our study results also showed that in-person health education had a significant impact on behavior changes among both intervention and control groups. The possible reasons can be, firstly, this study created scopes for Bangladeshi individuals as they have fewer opportunities to receive health education. Health resources are very limited in this country and people do not have a BP machine at home. Besides, they lack opportunity to check BP regularly due to inadequate health check-up systems. As we previously reported [45], people were aware of HTN when they have symptoms. In this study, CHWs have visited home to home and checked BP and food and urine salinity, which was a momentous opportunity for them. Thus, the home health care provided by CHWs is likely a major factor responsible for the improvement of outcomes. Second, the amount of salt intake on the previous day was measured at home using food salinity and urinary salinity devices, which showed the actual status of salt concentration, and participants could connect these data to their BP value.

Our results from the primary outcome showed the significant adherence differences only in salt intake and fruits intake behaviors between the intervention and control groups. Even though the differences were statistically significant, the salt intake change rate was more in the control group from baseline to program endpoint than in the intervention group. Compared to the baseline, almost no participants followed the target goal (<6 g daily salt intake) and this change was noteworthy. Concerning fruits intake behavior, the participants of the intervention group reached the target goal (fruits intake everyday/week) compared with the control group. Moreover, people ate vegetables every day in the rural community, but we did not inquire about the serving amount per day.

Regarding self-reported physical activity behaviors, both groups showed statistically significant differences. Physical activity is beneficial in weight reduction and also improves cardiovascular fitness [46]. Moderate to vigorous physical activity with a goal of 120-150 minutes on at least 5 days in a week should be encouraged [29].

Concerning self-reported BP and weight monitoring behaviors, in the first month (0.5 months and 1 month), participants answered “2 times/month,” because CHWs checked their BP and weight during their follow up. Then they reported “never/week” as none of the participants had facilities to measure these at home. This is possibly due to the cost of BP monitoring and weight-measuring devices, which is too exorbitant for the poor in Bangladesh. Moreover, BP monitoring requires the presence of a trained observer [47,48].

Supporting this self-report, the level of the salt concentration (1.2%-1.4% very thick) in the daily food (liquid) was reduced in both groups, more in the control group. With regard to urine salinity, the urinary salt excretion was reduced at the end of the study; however, it did not reach the recommended goal, and this finding is similar to other studies [49,50]. Combining primary and secondary outcomes, though the primary outcome variables were self-reported, the evidence from secondary outcome variables, which were quantifiable from the physical data, supported the positive changes of primary outcomes. Both SBP and DBP mean values were significantly decreased and SD became narrower in both groups.

As a comprehensive evaluation of this study, QOL of the participants was improved significantly after starting the intervention. This can be attributed to the reception of self-management health education, or the participants might have developed a better understanding of their illness, its effects, greater self-reported adherence, and a better relationship with the CHWs [51]. Thus, the implementation of our intervention created successfully more awareness among the participants.

### Strengths and Limitations

In this study, most of the female participants were housewives and thus available at home that allows them to play an influential role than males on behavior changes among the family members [52]. Females also seem to be more engaged, and apparently better-informed decision makers and more familiar in preparing foods, especially in rural areas [53]. This might be another reason for the good results observed on behavior change.

There were some limitations to this study. First, the primary outcome was self-reported by the participants. Second, we did not assess the household income impact as well as other influencing factors that may have had effects on diet among the participants. Third, this study was performed in the context of a small-scale program, and we chose purposive sampling which did not show the generalizability of our results. Fourth, most of the participants were female, and so we cannot generalize our study results to both genders. Fifth, data contamination was highly likely; therefore, the CHWs received verbal consent from the study participants not to disclose any study details with neighbors or other family members.

### Conclusions

The results of this study could not show the effectiveness of a combination of SMS text messaging and in-person health education; rather, in-person health education alone had a better impact on behavior changes. Based on the results of study, we suggest that in-person health education requires integration of home health care provision as a major factor for the improvement of outcomes as well as self-management behavior. Besides, it can be recommended that the more relevant and timely interactive SMS text messages can be sent to increase the effectiveness. Finally, the study recommends organizing community awareness meetings to create a “low-salt culture” and educate the family members. Moreover, further research in the diverse population living in different geographical areas is imperative to consolidate or refute our observation.

## Appendix

Multimedia Appendix 1 CONSORT-EHEALTH (V 1.6.1) -Submission/Publication Form.

Multimedia Appendix 2 Awareness development process and content of study outline (referred from reference 27).

Multimedia Appendix 3 Contents of text messages.

Multimedia Appendix 4 Behavioral change evaluation questionnaire.

Multimedia Appendix 5 Multinomial logit model.

Multimedia Appendix 6 Chronological behavior changes.

Multimedia Appendix 7 Awareness development process and contents of study outline (referred from reference 27).

## Abbreviations

BP: blood pressure
CHWs: community health workers
DASH: dietary approaches to stop hypertension
HTN: hypertension
NCDs: noncommunicable diseases
QOL: quality of life
RBS: random blood sugar
