# Supplementary figures and images for: Awareness Development and Usage of Mobile Health Technology Among Individuals With Hypertension in a Rural Community of Bangladesh: Randomized Controlled Trial
Source: J Med Internet Res. 2020 Dec 7;22(12):e19137. doi: 10.2196/19137 (PMC7752538; doi:10.2196/19137)

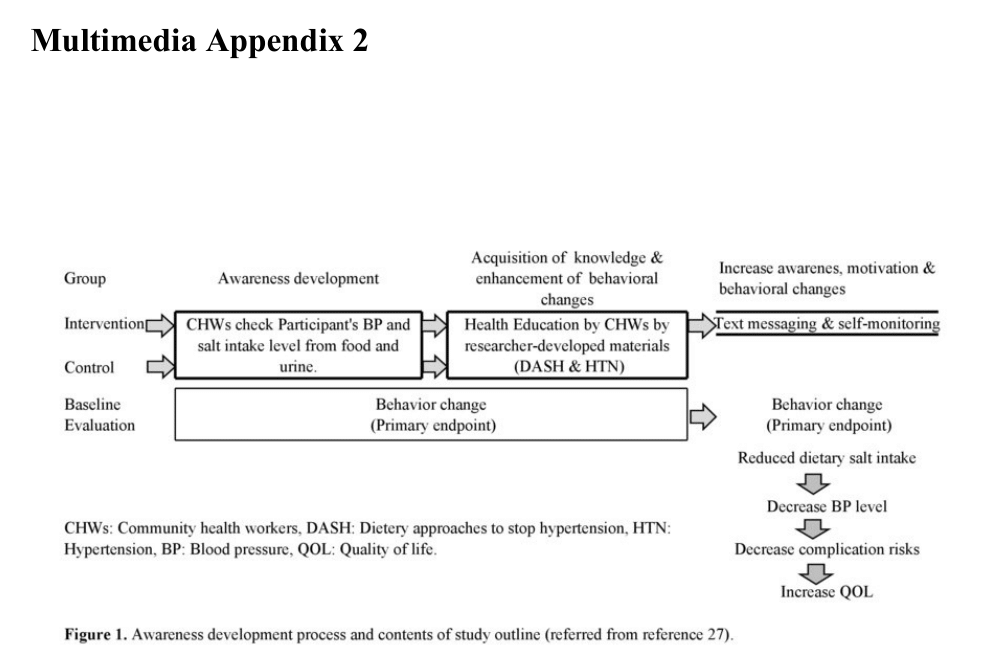

Supplement: Multimedia Appendix 2 [file jmir_v22i12e19137_app2.png]

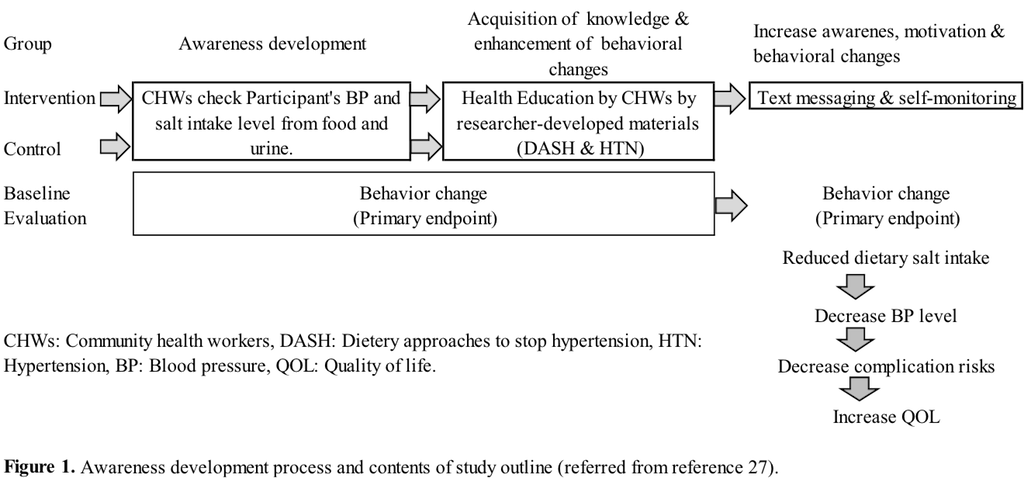

Supplement: Multimedia Appendix 7 [file jmir_v22i12e19137_app7.png]
