# Supplementary material for: Awareness Development and Usage of Mobile Health Technology Among Individuals With Hypertension in a Rural Community of Bangladesh: Randomized Controlled Trial
Source: J Med Internet Res. 2020 Dec 7;22(12):e19137. doi: 10.2196/19137 (PMC7752538; doi:10.2196/19137)
Supplement: Multimedia Appendix 3 [file jmir_v22i12e19137_app3.pdf]

Multimedia Appendix 3. Contents of text messages.

Message content	Examples	Text messaging schedules
Reduce sodium intake (salt)	Sodium intake is <6g/day (just under a teaspoon)<br>No extra salt, not even with fruits or green leafy vegetables, pickles, milk or any other foods<br>It keeps your high blood pressure under control	5 times for 1st month and once a week for the rest 4 months
Avoid taking oily and fatty foods	Like beef, mutton, poultry, Pastries, cakes and other junk foods<br>You can gain weight	
Eat more fruits and vegetables	Banana, starfruits, papaya and so on<br>80 grams per servings (fruits small size-full and large size-half) (per meal) per day<br>It makes you healthy	
Do exercise regularly	Regular exercise (walking, running, cycling, household work) for 30 minutes most days of the week<br>It keeps your heart well	
Take your medicine regularly	Don't change and stop your hypertensive treatment without your doctor's guidance	

Examples of two-way text messaging component

CHWs: Reduce salt intake.
Participant A: How much salt I need to cut?
CHWs: You can measure like under a teaspoon.
Participant A: Ok, I will.

CHWs: Avoid taking oily and fatty foods.
Participant B: What kind of foods should I stop?
CHWs: Beef, mutton, pastries!
Participant B: Ok.

CHWs: Eat more fruits and vegetables.
Participant C: What types of fruits and vegetables?
CHWs: Banana, guava, green leafy vegetables!
Participant C: Ok.

CHWs: Do exercise regularly.
Participant D: What types and how long?
CHWs: Walking, household work at least 30 minutes daily.
Participant D: I am doing, but not every day.
CHWs: Please do it every day.
Participant D: I will follow.

CHWs: Don't change or stop your medicine without your doctor's guidance.
Participant E: I drop it sometimes.
CHWs: Please don’t do that.
Participant E: Ok.
