# Supplementary material for: Awareness Development and Usage of Mobile Health Technology Among Individuals With Hypertension in a Rural Community of Bangladesh: Randomized Controlled Trial
Source: J Med Internet Res. 2020 Dec 7;22(12):e19137. doi: 10.2196/19137 (PMC7752538; doi:10.2196/19137)
Supplement: Multimedia Appendix 4 [file jmir_v22i12e19137_app4.pdf]

**Multimedia appendix 4.** Behavioral change evaluation questionnaire.

| | Questions | 5 | 4 | 3 | 2 | 1 |
|---|---|---|---|---|---|---|
| 1. | How many days do you eat fruits in a week? | Everyday/week | 5-6 days/week | 3-4 days/week | 1-2 days/week | 0 day/week |
| 2. | How many days do you eat vegetables in a week? | Everyday/week | 5-6 days/week | 3-4 days/week | 1-2 days/week | 0 day/week |
| 3. | How much salt do you take per day in a week (goal is <6g/day)? | Everyday/week (<6g daily intake) | 5-6 days/week (approx. 7-8g daily intake) | 3-4 days/week (approx. 9-10g daily intake) | 1-2 days/week (approx. 11-12g daily intake) | 0 day/week (approx. 13-14g daily intake) |
| 4. | How many days do you do 30 minutes physical activity/exercise in a week? | Everyday/week | 5-6 days/week | 3-4 days/week | 1-2 days/week | 0 day/week |
| 5. | How frequently do you check your blood pressure in a month? | 8 times/month | 6 times/month | 4 times/month | 2 times/month | Never/ month |
| 6. | How frequently do you monitor your body weight in a month? | 8 times/month | 6 times/month | 4 times/month | 2 times/month | Never/ month |
