# Supplementary material for: Awareness Development and Usage of Mobile Health Technology Among Individuals With Hypertension in a Rural Community of Bangladesh: Randomized Controlled Trial
Source: J Med Internet Res. 2020 Dec 7;22(12):e19137. doi: 10.2196/19137 (PMC7752538; doi:10.2196/19137)
Supplement: Multimedia Appendix 5 [file jmir_v22i12e19137_app5.pdf]

Multimedia Appendix 5 about the multinomial logit model: Figure 2, Table S1-S2

1-1. Terms for the segmentation and the model selection

As we described in the manuscript, salt intake, fruits intake, vegetables intake, physical activity, blood pressure monitoring, and body weight monitoring behavior changes were investigated. The trend in the behavior change was not constant in salt intake, physical activity, blood pressure monitoring, and body wight monitoring behaviors. For the segmented multinomial logit model, we defined the terms for segmentation which shown in Figure S2.

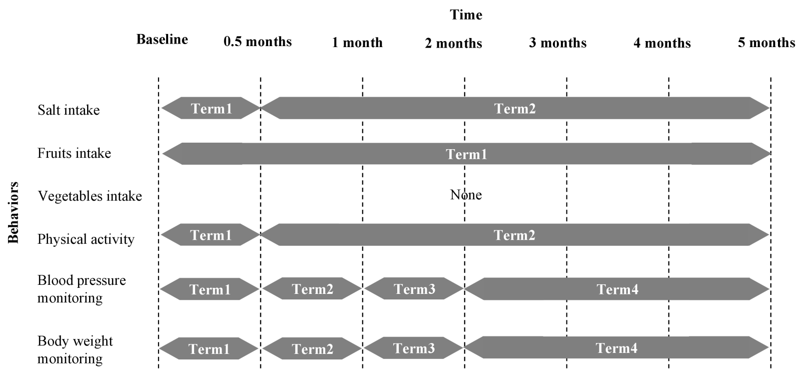

Figure S2. Segmentation time in the multinomial logit model for each behavior.

1-2. Salt intake

Likert-type response scale score 5 existed at baseline but disappeared from 0.5 months to 5 months in both groups. After decreasing the Likert-type response scale score 4 from baseline and 0.5 months, the increasing trends were observed on Likert-type response scale scores of 2 to 4 among both groups. Although a slight decrease of Likert-type response scale score 4 between 1 month and 2 months was found, we assumed the constant changes of both groups in term 1 and term 2. According to the result of the likelihood ratio test, we selected the Time-Group model as a suitable model, and the coefficients were obtained (Table S1). As shown, it is not significant in Group coefficients and there was no Group effect in term 1. On the contrary, the Group coefficients for Likert-type response scale scores 3 and 4 showed significant negative values. This meant that the intervention negatively affected those categories compared to the control. Taking the exponential of estimated “Group” coefficients on Likert-type response scale score 3 and score 4 in the term 2, the relative effects were estimated as 0.63 (95% CI; 0.50-0.79) times decrease in score 3 and 0.29 (95% CI; 0.22-0.39) times decrease in score 4.

Table S1. Estimates and the significance obtained by multinomial logit model for salt intake behavior.

Term 1 (Baseline-0.5 months)						
Likert-type response scale score	Intercept (95% CI)	*P-value*	Time (95% CI)	*P-value*	Group (95% CI)	*P-value*
1 (Reference)<br>0 day/week (approx. 13-14g daily intake)	-	-	-	-	-	-
2<br>1-2 days/week (approx. 11-12g daily intake)	-0.83<br>(-1.14, -0.53)	<.001	-0.58 NS<br>(-1.30, 0.14)	.11	-0.10 NS<br>(-0.46, 0.26)	.57
3<br>3-4 days/week (approx. 9-10g daily intake)	-0.96<br>(-1.28, -0.64)	<.001	-0.67 NS<br>(-1.41, 0.07)	.07	0.06 NS<br>(-0.31, 0.43)	.75
4<br>5-6 days/week (approx. 7-8g daily intake)	-1.57<br>(-2.02, -1.12)	<.001	-4.69<br>(-6,44, -2.95)	<.001	0.29 NS<br>(-0.29, 0.87)	.33
5<br>Everyday/week (<6g daily intake)	-3.94<br>(-5.35, -2.54)	<.001	-15.19 NS<br>(-92.92, 62.55)	.70	0.03 NS<br>(-1.95, 2.02)	.97
Term 2 (0.5-5 months)						
Likert-type response scale	Intercept	*P-value*	Time	*P-value*	Group	*P-value*
1 (Reference)<br>0 day/week (approx. 13-14 g daily intake)	-	-	-	-	-	-
2<br>1-2 days/week (approx. 11-12 g daily intake)	-0.94<br>(-1.17, -0.71)	<.001	0.51<br>(0.43-0.60)	<.001	-0.13 NS<br>(-0.36, 0.10)	.25
3<br>3-4 days/week (approx. 9-10 g daily intake)	-1.41<br>(-1.65, -1.17)	<.001	0.81<br>(0.72, 0.90)	<.001	-0.47<br>(-0.70, -0.24)	<.001
4<br>5-6 days/week (approx. 7-8 g daily intake)	-2.75<br>(-3.08, -2.42)	<.001	1.16<br>(1.05, 1.27)	<.001	-1.23<br>(-1.52, -0.94)	<.001
5<br>Everyday/week (<6 g daily intake)	-	-	-	-	-	-

NS: not significant, and the estimates were regarded as not apart from zero.

1-3. Fruits intake

We found a consistent increasing trend of the proportion of Likert-type response scale score 5 except from 2 months to 3 months (Figure S3). Though higher rate of increasing trend was observed from Likert-type response scale score of 2 to 4 between baseline to 0.5 months, we assumed constant time effect for the proportion of each Likert-type response scale score. According to the result of the likelihood ratio test, we selected the Time-Group model as suitable one, and the estimates of coefficients were obtained (Table S2). As shown in the estimated positive values, the effect of Group "intervention" contributed to the increasing trend of Likert-type response scale score of 2 to 5. Taking the exponential of estimated "Group" coefficients, the relative effects were estimated as 1.28 (95% CI; 1.00-1.61) times increase in score 2, 1.40 (95% CI; 1.11-1.78) times increase in score 3, 2.07 (95% CI; 1.50-2.85) times increase in score 4, and 2.24 (95% CI; 1.68-2.98) times increase in score 5.

Table S2. Estimates and the significance obtained by multinomial logit model for fruits intake behavior.

Likert-type response scale	Intercept (95% CI)	*P-value*	Time (95% CI)	*P-value*	Group (95% CI)	*P-value*
1 (Reference) 0 day/week	-	-	-	-	-	-
2 1-2 days/week	0.28 (0.08, 0.48)	.007	0.20 (0.12, 0.28)	<.001	0.24 (0.00, 0.48)	.040
3 3-4 days/week	-0.07NS (-0.28, 0.14)	.490	0.39 (0.32, 0.47)	<.001	0.34 (0.10, 0.58)	.005
4 5-6 dyas/week	-1.67 (-1.99, -1.36)	<.001	0.43 (0.33, 0.53)	<.001	0.73 (0.40, 1.05)	<.001
5 Everyday/week	-1.53 (-1.99, -1.36)	<.001	0.53 (0.44, 0.62)	<.001	0.80 (0.52, 1.09)	<.001

NS: not significant and the estimates were regarded as not apart from zero.

1-4. Vegetables intake

Through all the study periods, only Likert-type response scale score 5 was observed in the control group as well as in the intervention group except 2 participants of Likert-type response scale score 4. The participants routinely ate vegetables everyday. We did not observe the behavior change in both groups. Therefore, no statistical analysis was implemented in this behavior.

1-5. Physical activity

We observed a sudden increase of Likert-type response scale score 5 from baseline to 0.5 months, and Likert-type response scale score 5 gradually increased after the point in both groups. According to the result of the likelihood ratio test (*P*=.36), we selected the Time-only model (Table 3). We did not show the result of estimation because our purpose was to measure the difference between the Intervention group and Control group.

1-6. Blood pressure monitoring

Similar trend was observed in both intervention and control groups, after an increase of Likert-type response scale score 2 from baseline to 0.5 months, a decrease was observed between 1 month to 2 months. From 2 months, some participants kept the Likert-type response scale score 2. According to the result of the likelihood ratio test (*P*=.41), we selected the Time-only model (Table 3).

1-7. Body weight monitoring

After an increase of Likert-type response scale score 2 from baseline to 0.5 months, a decrease was observed between 1 month to 2 months in both groups. From 2 months, the proportion of Likert-type response scale score 2 slightly decreased in the intervention group but almost kept around 3% in the control group. According to the result of the likelihood ratio test (*P*=.32), we selected the Time-only model (Table 3).
