# Supplementary material for: Awareness Development and Usage of Mobile Health Technology Among Individuals With Hypertension in a Rural Community of Bangladesh: Randomized Controlled Trial
Source: J Med Internet Res. 2020 Dec 7;22(12):e19137. doi: 10.2196/19137 (PMC7752538; doi:10.2196/19137)

Multimedia Appendix 6

Chronological behavior changes: Figure S3 to S7

1. Fruits intake (Figure S3)

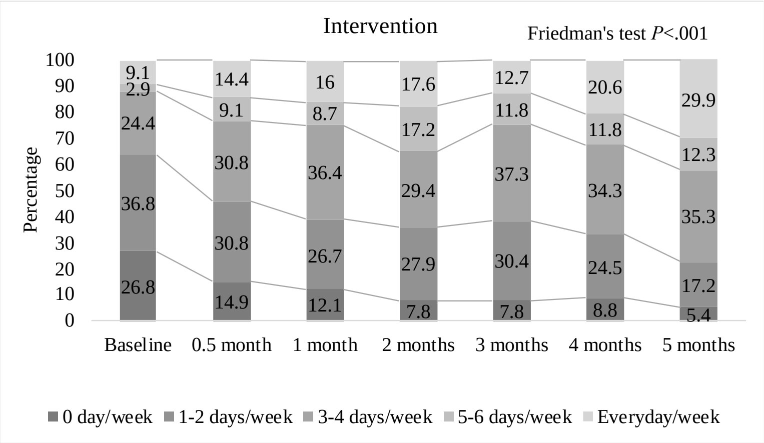

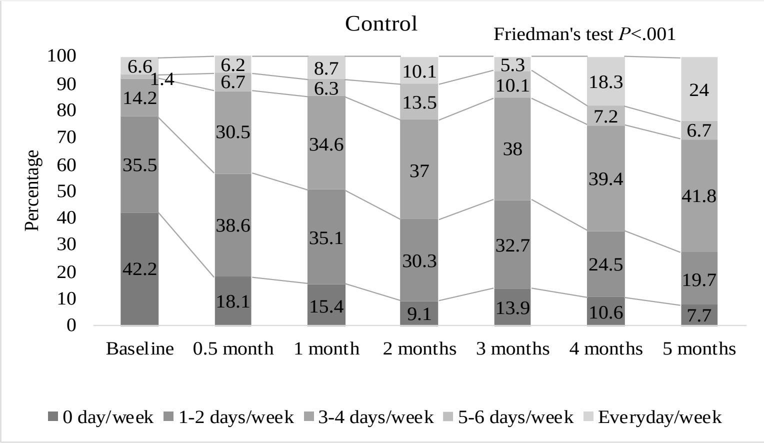

2. Vegetables intake (Figure S4)

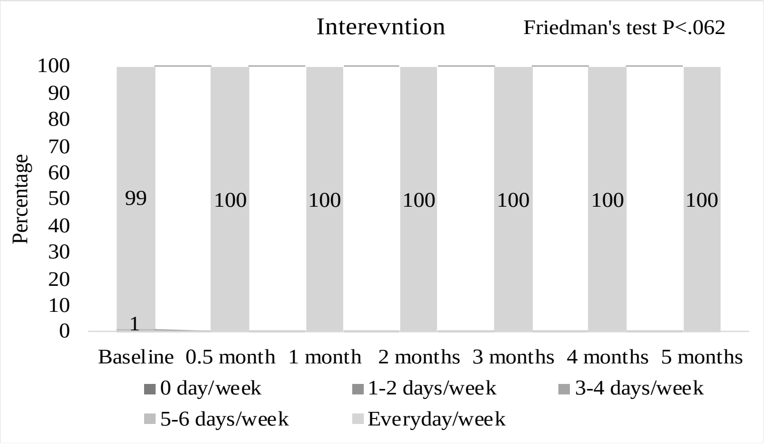

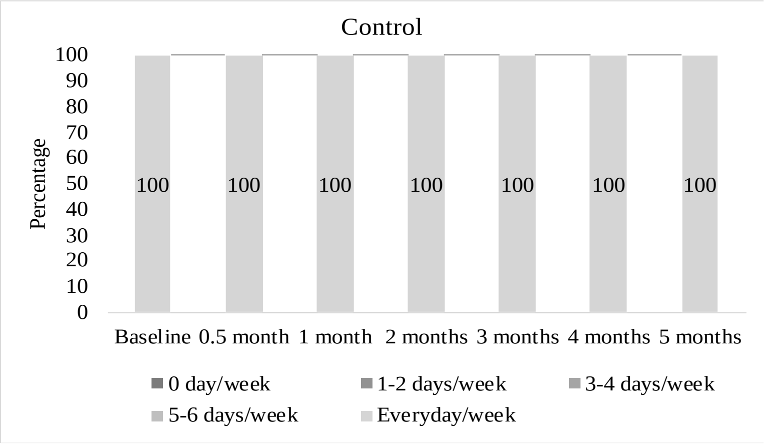

3. Physical activity (Figure S5)

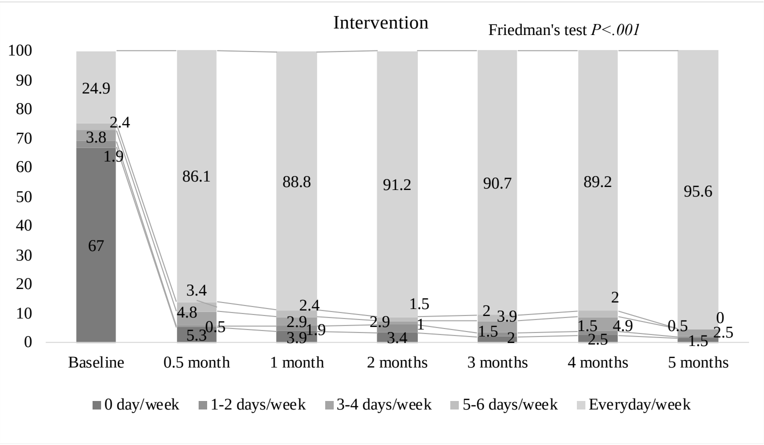

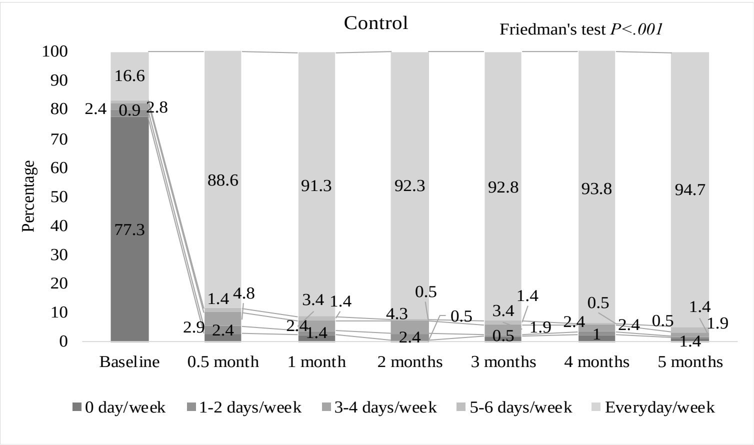

4. Blood pressure monitoring (Figure S6)

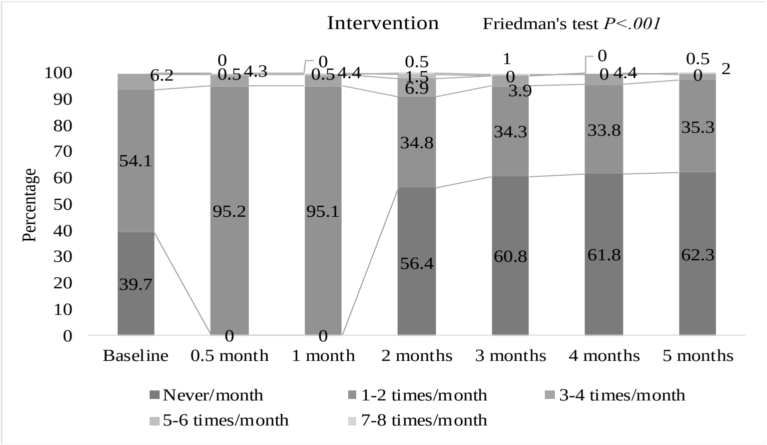

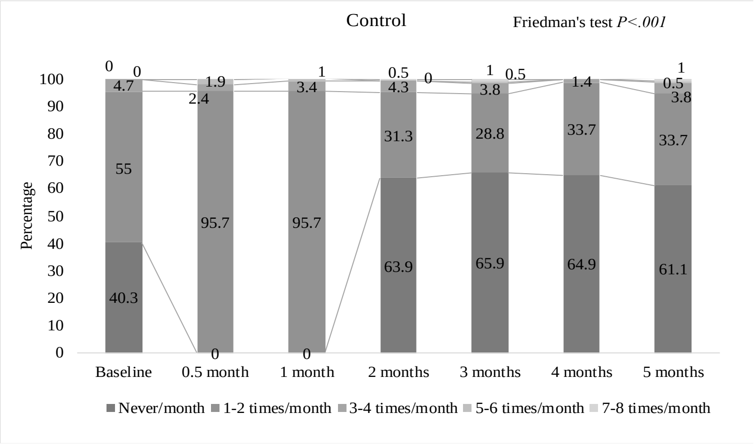

5. Body weight monitoring (Figure S7)

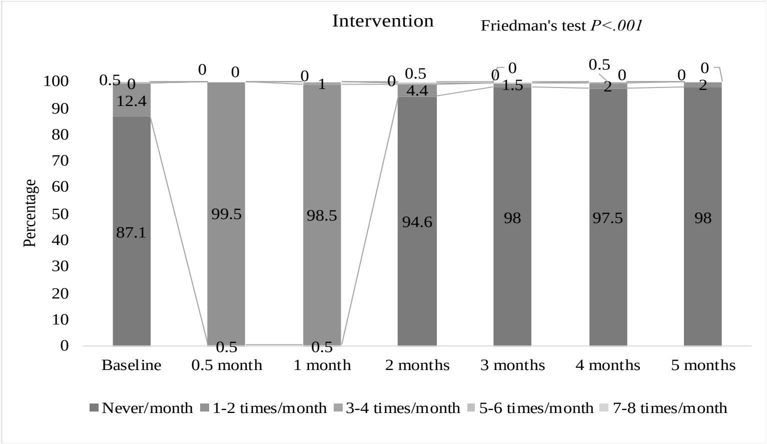

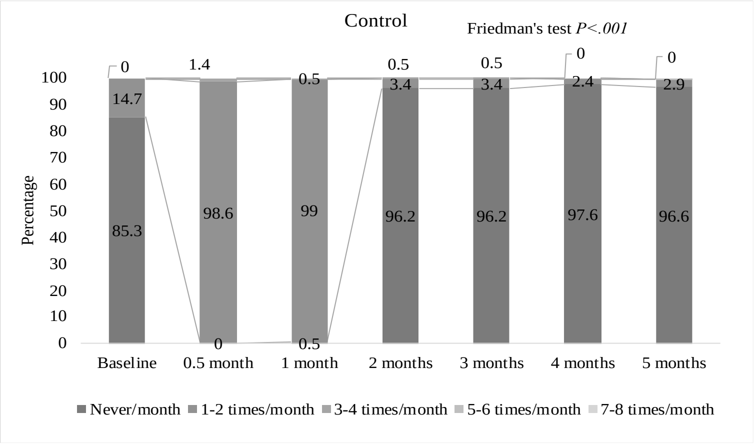

Supplement: Multimedia Appendix 6 [file jmir_v22i12e19137_app6.pdf]
